# Pharmakokinetische und metabolische Besonderheiten der Antibiotikaanwendung bei Kindern und älteren Menschen

**DOI:** 10.1007/s00103-026-04233-7

**Published:** 2026-04-13

**Authors:** Nils-Olaf Hübner

**Affiliations:** Institut für Hygiene und Umweltmedizin mit Zentralbereich Hygiene, Universitätsmedizin Greifswald, Walter-Rathenau-Str. 49, 17489 Greifswald, Deutschland

**Keywords:** Antiinfektivatherapie, Pharmakokinetik/Pharmakodynamik, Alte Menschen, Kinder, Resistenzentwicklung, Anti-infective therapy, Pharmacokinetics/pharmacodynamics (PK/PD), Elderly, Children, Antimicrobial resistance

## Abstract

Die Wirksamkeit antiinfektiver Therapien beruht auf dem Erreichen eines pharmakokinetischen/pharmakodynamischen Ziels. Daher ist für die Wirksamkeit und Sicherheit antiinfektiver Therapien die korrekte Dosierung entscheidend. Dieser Artikel gibt einen Überblick über die pharmakologischen Besonderheiten der Antiinfektivatherapie bei älteren Menschen und Kindern. Insbesondere für diese Gruppen besteht ein erhöhtes Risiko für Fehldosierungen, da standardisierte Dosierungsschemata in der Regel auf Studien mit Erwachsenen mittleren Alters basieren und Personen mit abweichender Pharmakokinetik (Aufnahme, Verteilung, Verstoffwechselung und Ausscheidung; PK) und Pharmakodynamik (Wechselwirkung mit dem Erreger und therapeutische Wirkung; PD) mitunter nicht gut abbilden. Fehldosierungen erhöhen jedoch die Wahrscheinlichkeit für ein Therapieversagen, die Entwicklung antimikrobieller Resistenzen und Nebenwirkungen.

Bei älteren Menschen können verminderte Resorption, veränderte Körperzusammensetzung, reduzierte Leber- und Nierenfunktion sowie Polypharmazie die Wirkstoffaufnahme, -verteilung, -metabolisierung und -elimination beeinflussen. Bei Kindern spielen altersabhängige Unterschiede in der Organreifung, Enzymaktivität und glomerulären Filtrationsrate eine Rolle. Diese Besonderheiten können den Therapieerfolg beeinträchtigen und bei subtherapeutischen Dosierungen zur Selektion resistenter Mikroorganismen führen.

Für Ältere und Kinder ist daher eine individualisierte Dosisanpassung unter Berücksichtigung der pharmakokinetischen Besonderheiten erforderlich. Zudem sind eine gute Aufklärung und Begleitung essenziell, um Wirksamkeit, Sicherheit und Adhärenz zu gewährleisten und die Verbreitung von Resistenzen zu vermeiden.

## Abstract (en)

The efficacy of antimicrobial therapies depends on achieving a pharmacokinetic/pharmacodynamic target. Therefore, correct dosing is crucial for the efficacy and safety of antimicrobial therapies. This article provides an overview of the pharmacological considerations of antimicrobial therapy in older adults and children. These groups, in particular, face an increased risk of improper dosage, as standardized dosing regimens are generally based on studies in middle-aged adults and may not adequately reflect individuals with different pharmacokinetics (absorption, distribution, metabolism, and excretion; PK) and pharmacodynamics (interaction with the pathogen and therapeutic effect; PD). However, incorrect dosing increases the likelihood of treatment failure, the development of antimicrobial resistance, and side effects.

In older adults, absorption, distribution, metabolism, and elimination of the anti-infective agent can be affected by reduced absorption, changes in body composition, reduced liver and kidney function, and polypharmacy. In children, relevant aspects are age-related differences in organ maturation, enzyme activity, and glomerular filtration rate. These factors can impair treatment success and can lead to subtherapeutic doses, thus fostering selection of resistant microorganisms.

For older adults and children, individualized dose adjustments that take into account their pharmacokinetic specifics are therefore necessary. In addition, thorough patient education and support are essential to ensure efficacy, safety, and adherence as well as to prevent the spread of resistance.

## Einleitung

Die Problematik der Resistenz gegen Antiinfektiva ist so alt wie die Antiinfektiva selbst. Schon Alexander Fleming ging in seiner Nobelpreisansprache auf das Problem ein: „Es ist nicht schwer, Mikroorganismen im Labor gegen Penicillin resistent zu machen, indem man sie Konzentrationen aussetzt, die nicht ausreichen, um sie abzutöten. Gleiches ist auch manchmal schon im Körper passiert. Es wird vielleicht die Zeit kommen, da jeder Penicillin kaufen kann. Dann besteht die große Gefahr, dass der Unwissende die Dosis zu gering wählt, die Erreger so nicht-tödlichen Konzentrationen aussetzt und sie so resistent macht“ [1].

Diese Sätze sind heute so aktuell wie damals, auch wenn sich das Wissen dazu deutlich erweitert hat: Die Kernpunkte sind

- erstens die Entwicklung oder Selektion resistenter Stämme bei Exposition gegen Konzentrationen antimikrobieller Wirkstoffe, die nicht ausreichen, um die Erreger abzutöten;
- zweitens, dass dies zügig und sowohl im Labor als auch in klinischen Anwendungen geschehen kann; und
- drittens, dass dies unbeabsichtigt durch Fehldosierungen aufgrund falscher Annahmen passieren kann.

Vor allem ältere Menschen und Kinder sind in klinischen Studien oft unterrepräsentiert. [2–6]. Gerade bei Älteren kann es dadurch zu Fehldosierungen kommen, insbesondere wenn sie schlicht als „Erwachsene“ (im Gegensatz zu Kindern) eingestuft und Applikationsschemata unreflektiert übertragen werden.

Im Jahr 2022 machten Ältere (über 65 Jahre) 23 % der Bevölkerung in Deutschland aus und gehörten zu den Hauptkonsumenten von Arzneimitteln [7–9]. Besonders die Gruppe der Hochbetagten (ab 85 Jahren) ist in den letzten Jahren überproportional gewachsen: Sie zählte 1991 noch knapp 1,2 Mio. und stieg bis 2024 insgesamt auf 3,0 Mio. Personen an [9]. Der Antibiotikaeinsatz in der Gruppe der über 65-Jährigen liegt deutlich höher als bei jüngeren Erwachsenen [10, 11]. Das liegt in einer höheren Infektionsanfälligkeit und -häufigkeit bei Älteren begründet [12]. Höhere Altersgruppen tragen zudem ein im Vergleich zur mittleren Altersgruppe gesteigertes Risiko für Infektionen mit multiresistenten Erregern [13]. Mit dem Alter steigen zudem Multimorbidität, Polypharmazie und funktionelle Defizite an [7, 14]. Hinzu kommen Veränderungen der Physiologie, die die Pharmakokinetik (das, was der Körper mit dem Arzneistoff tut, d. h. Aufnahme, Verteilung, Verstoffwechselung und Ausscheidung) und damit die Wahrscheinlichkeit der Erreichung der Zielspiegel von Antiinfektiva beeinflussen können [15–19].

Mit dem Alter steigt zudem der Anteil Pflegebedürftiger: Ende 2023 waren rund 11 % der 70- bis 74-Jährigen pflegebedürftig, während der Anteil bei den über 90-Jährigen bei 87 % lag. Von den insgesamt 5,69 Mio. Pflegebedürftigen wurden 14 % in Pflegeheimen vollstationär betreut [20, 21]. Auch hier zeigt sich eine deutliche Altersabhängigkeit: In der Gruppe der 65- bis 70-Jährigen wurden 11,8 % vollstationär gepflegt, bei 80- bis 85-Jährigen 14,2 % und bei den über 95-Jährigen 37,3 %. Bei Kindern unter 15 Jahren waren es lediglich 0,2 %. Gerade in Pflegeeinrichtungen sind Antiinfektivatherapien und antimikrobielle Resistenzen häufig: Die aktuelle europäische Healthcare-associated Infections in European Long-Term Care Facillities(HALT)-4-Studie (2024) zeigt für Deutschland eine mediane Prävalenz von 0,9 % für mindestens eine antibiotische Therapie. Damit liegt die Prävalenz bei Bewohnern in Pflegeeinrichtungen deutlich über dem Mittelwert für Personen in den Altersgruppen 65+ (0,5 %–0,6 %; [8, 22]).

Die Zusammenhänge zwischen Alter und Resistenz sind komplex. Eine patientenbezogene Auswertung der Daten des „European Antimicrobial Resistance Surveillance Network (EARS-NeT)“ zu Blutstrominfektionen 2015–2019 zeigte substanzielle Unterschiede in der Resistenz zwischen und innerhalb der eingeschlossenen 29 Länder. Die Autoren kommen zu dem Schluss, dass Alter und Geschlecht wichtige Faktoren sind und z. B. dass Ältere, insbesondere Männer, stärker von Infektionen mit resistenten Bakterien betroffen, die genauen Interaktionen aber unbekannt sind [7, 8, 12, 14–19, 22–24].

Dieser Artikel gibt zunächst einen Überblick über die Grundlagen der Wirksamkeit von Antiinfektiva mit Fokus auf Antibiotika und geht dann auf die pharmakokinetischen Besonderheiten bei älteren Menschen und Kindern ein. Abschließend werden der Einsatz von Antiinfektiva und die dafür notwendigen Kenntnisse, gerade bei Älteren und Kindern, diskutiert.

## Grundlagen der Wirksamkeit, Pharmakokinetik (PK) und Pharmakodynamik (PD) von Antiinfektiva

Aussagen zur Wirksamkeit und Dosierung von Antiinfektiva basieren neben der mikrobiologischen (d. h. *in vitro* bestimmten) minimalen Hemmkonzentration (MHK, MIC) wesentlich auf ihren pharmakologischen und pharmakokinetischen Parametern. Diese bestimmen letztlich das Schicksal der Substanzen nach ihrer Applikation (Pharmakokinetik, PK) sowie ihre Fähigkeit, ihre Wirkung auf den Zielorganismus (Pharmakodynamik, PD) unter klinischen Bedingungen, d. h. im Körper, zu erreichen [19, 25]. Die optimale Dosierung (Menge, Dauer, Intervall) ist dabei jene, die mit möglichst hoher Wahrscheinlichkeit eine Hemmung oder Abtötung des Erregers sowie eine klinische Heilung erzielt.

**Abb. 1:**
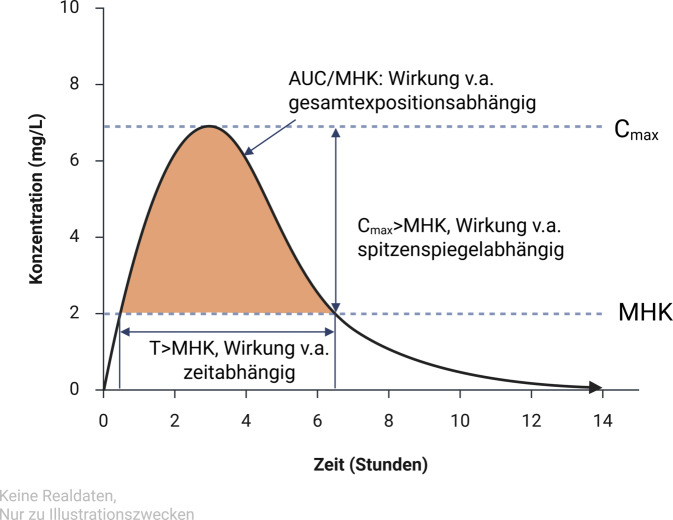
PK/PD-Indizes für Antiinfektiva. Schematische Darstellung des Konzentrationsverlaufs im Organismus. (*AUC* Area-under-the-Curve, *C*_*max*_ Spitzenkonzentration, *MHK* minimale Hemmkonzentration, *PD* Pharmakodynamik, *PK* Pharmakokinetik, *T* Zeit)

Pharmakokinetik und Pharmakodynamik lassen sich zueinander ins Verhältnis setzen, indem PK/PD-Indizes gebildet werden. Als besonders hilfreich haben sich dabei erwiesen:

1. die Zeit (bzw. der Anteil des Dosierungsintervalls), während die Konzentration des Antiinfektivums über der MHK liegt (T > MHK),
2. die Spitzenkonzentration des Antiinfektivums im Vergleich zur MHK (C_max_ > MHK) sowie
3. die Summe der Konzentrations-Zeit-Relationen innerhalb von 24 h oberhalb der MHK (Gesamtexposition, Area-under-the-Curve, AUC/MHK; [25]; Abb. 1).

Die Aktivität verschiedener Klassen von Antiinfektiva lässt sich jeweils besonders gut mit einem dieser Indizes beschreiben: So gehören Aminoglykoside, Polymyxine, Metronidazol (auch Daptomycin und Fluorochinolone, s. unten), zu den Antiinfektiva, deren Wirkung v. a. über ihren Spitzenspiegel (Cmax > MHK; und auch ihre AUC/MHK) beschrieben werden kann. Das ist u. a. darauf zurückzuführen, dass sie einen ausgeprägten postantibiotischen Effekt aufweisen, d. h. die Hemmwirkung auch nach dem Abfall der Konzentration unter die MHK weiterhin fortbesteht [25].

Im Gegensatz dazu ist die Wirksamkeit z. B. vieler β‑Laktam-Antibiotika wie Penicilline, Cephalosporine, Carbapeneme oder Monobactame nicht primär vom Spitzenspiegel, sondern v. a. von der Zeit abhängig, während der ihre Konzentration über der MHK liegt (T > MHK). Sie weisen eine geringe postantibiotische Aktivität auf [25].

Die Wirksamkeit von Antibiotika, die – ähnlich wie die erste Gruppe – eine deutliche postantibiotische Aktivität aufweisen, deren Wirkung aber mehr von der erreichten Gesamtexposition über 24 h abhängt als von der Maximalkonzentration, lässt sich als dritte Gruppe v. a. über die AUC/MHK und in zweiter Linie über die Cmax/MHK beschreiben. Zu dieser Gruppe gehören beispielsweise Tetracycline, Tigecyclin, Makrolide, Azithromycin, Linezolid, Trimethoprim, Sulfonamide oder Vancomycin (auch Daptomycin, Clindamycin und Fluorchinolone s. unten; [25–29]).

Eine wirksame antiinfektive Therapie basiert daher auf dem Erreichen eines pharmakodynamischen Ziels („pharmacodynamic-pharmacokinetic target“, PK/PD-target). Das PK/PD-target stellt den minimal angestrebten PK/PD-Index dar, bei dem mit hoher Wahrscheinlichkeit ein Behandlungserfolg erzielt wird (z. B. das minimale Verhältnis von Spitzenspiegel zu MHK). Dies gilt nicht nur für den Patienten insgesamt, sondern auch für das Zielkompartiment, das für die Therapie erreicht werden muss [27, 28, 30].

Die Wahrscheinlichkeit, das PK/PD-target zu erreichen, hängt sowohl von der Dosierung als auch der Pharmakokinetik des Antiinfektivums ab. Die Pharmakokinetik ist individuell verschieden und auch intraindividuell nicht konstant. Dosierungen von Antiinfektiva sind jedoch standardisiert. Daraus ergibt sich, dass bei Personen mit vom Durchschnitt abweichender Pharmakodynamik der Therapieerfolg ggf. nicht den Erwartungen entspricht, sofern dies nicht bei der Therapie berücksichtigt wird, etwa durch Anpassung von Dosis, Dauer und Applikationsweg. Obwohl Daten zur individuellen Pharmakokinetik rar sind und z. B. ein therapeutisches Drug-Monitoring (d. h. die wiederholte Messung der Arzneistoffkonzentration im Körper während der Therapie) nicht überall und nicht für alle Antiinfektiva zur Verfügung steht, liegen für größere Personengruppen wie Kinder und ältere Menschen Daten vor, die eine gezielte Anpassung der Dosierung unterstützen können (s. unten; [27, 29]).

## Pharmakokinetische Besonderheiten bei älteren Menschen

Lebensaltersassoziierte Änderungen der Physiologie können auch die Pharmakokinetik von Antiinfektiva verändern. Zu den durch die Alterungsprozesse selbst bedingten Effekten kommen ggf. direkte und indirekte Interaktionen durch andere Medikamente, d. h. durch Polypharmazie bedingte Veränderungen, hinzu.

### Aufnahme (Absorption).

Höheres Alter ist mit Änderungen der Absorption aus dem Gastrointestinaltrakt assoziiert. Das beginnt bereits im Mund (verstärkte Neigung zur Mundtrockenheit) und setzt sich mit tendenziell reduzierter Motilität des Ösophagus, des Magens und Dünndarms fort. Infolge ist die Magen-Darm-Passage verlängert. Durch verminderte Kapazität zur Säurebildung ist zudem der Magen-pH-Wert tendenziell erhöht [19, 31]. Die Expression wichtiger Transportproteine kann vermindert sein [25]. Das kann zu Veränderungen der Absorption peroral verabreichter Antiinfektiva führen. So sinkt potenziell die Absorption von z. B. Azithromycin, Erythromycin, Cefaclor, Ceftibuten, Cefpodoxime, Sulfonamiden, aber auch von Itraconazol und Ketoconazol. Diese Effekte können z. B. durch die Gabe von Protonenpumpeninhibitoren (PPI) noch weiter verstärkt werden [15].

### Verteilung (Distribution).

Im Alter kommt es allgemein zur Abnahme der mageren Körpermasse und des Gesamtwasseranteils bei gleichzeitiger Zunahme des Fettgehalts sowie einer Tendenz zur Hypoalbuminämie. Daraus können sich Veränderungen des Verteilungsvolumens sowie der Plasmakonzentrationen und Halbwertszeiten von Antiinfektiva ergeben. Ein erhöhter Körperfettanteil führt zu einem größeren Verteilungsvolumen für lipophile Wirkstoffe wie Fluorchinolonen, Makroliden oder Rifampicin. Bei Hypoalbuminämie kann der Anteil freier Wirkstoffe (insbesondere saurer Substanzen wie Penicillinen, Ceftriaxone, Sulfonamiden oder Clindamycin) erhöht sein. Zudem kann die im Alter tendenziell reduzierte kardiovaskuläre Leistungsfähigkeit zu Änderungen der Perfusion und vermehrter (lokaler) Flüssigkeitseinlagerung im Gewebe führen. In der Folge kann es zu einer reduzierten Konzentration vor allem hydrophiler Wirkstoffe am Ort der Infektion kommen [15, 25]. Diese theoretisch ableitbaren Änderungen sind als möglich, aber nicht immer gegeben zu erwarten: So kam z. B. eine systematische Übersichtsarbeit zur Pharmakokinetik von β‑Laktamen zu dem Schluss, dass das Verteilungsvolumen bei Älteren nicht generell erhöht ist, obwohl dies bei β‑Laktamen als hydrophile Substanzen zu erwarten wäre [16]. Als mögliche Erklärungen werden angeführt, dass Hochbetagte (> 85 Jahren) in Studien häufig unterrepräsentiert sind und Gebrechlichkeit (Frailty) – und nicht Alter an sich – wesentlich für eine veränderte Pharmakokinetik ist [16].

### Verstoffwechselung (Metabolismus).

Ein durch eine reduzierte Leberfunktion bedingter verminderter First-Pass-Effekt (Wirkstoffelimination bei der ersten Leberpassage) kann sich je nach Substanz unterschiedlich auswirken: Einerseits kann die Elimination vermindert sein, andererseits kann die Kapazität zur Aktivierung von Prodrugs, die erst in der Leber aktiviert werden, wie z. B. bei Metronidazol, reduziert sein [25]. Aber auch nach dem First-Pass oder bei intravenös verabreichten Substanzen haben altersbedingte Änderungen der Stoffwechselleistung Auswirkungen auf den Metabolismus von Antiinfektiva und können die Pharmakokinetik verändern. Hepatisch verstoffwechselte Medikamente, die gleichzeitig gegeben werden, haben durch Induktion einerseits und Kompetition/Inhibition andererseits (zum Beispiel des Cytochrome-P450-Systems) ein entsprechend hohes Interaktionspotenzial [17, 19, 25].

### Ausscheidung (Elimination).

Höheres Alter ist mit einer Verminderung der Nierenfunktion assoziiert. In der Folge kommt es zur Reduktion der glomerulären Filtrationsrate (GFR) und zur verlängerten Eliminationshalbwertszeit, insbesondere für renale Antibiotika (wie z. B. Aminopenicilline, Aminoglykoside, Chloramphenicol, Gyrasehemmer, Tetracycline, Vancomycin; [17, 25]).

## Pharmakologische Besonderheiten bei Kindern

Kinder weisen eine im Vergleich zu Erwachsenen stark vom Lebensalter abhängig veränderte Physiologie auf. Das kann auch Einfluss auf die Pharmakokinetik von Antiinfektiva haben. Umfassende Übersichten, auch zur Pharmakokinetik einzelner Substanzen, finden sich in den Quellen.

### Aufnahme (Absorption).

Der pH-Wert des Magens reifgeborener Kinder sinkt nach der Geburt zunächst stark ab, um nach ca. 8–10 Tagen wieder in einen neutralen Bereich zu steigen und dann innerhalb der ersten 2–3 Lebensjahre auf ein Niveau zu sinken, das mit dem von Erwachsenen vergleichbar ist. Bei Frühgeborenen kann diese Dynamik abgeschwächt sein [30]. Bei höherem Magen-pH werden säurelabile Antibiotika (z. B. Benzylpenicillin (Penicillin G), Ampicillin, Amoxicillin, Flucloxacillin und Erythromycin) in geringerem Ausmaß inaktiviert mit der Folge höherer Bioverfügbarkeit und Plasmaspiegel [32]. Ebenso ist die Geschwindigkeit der Magenpassage bei Neugeborenen vermindert (bis zu 6–8 h) und erreicht erst nach ca. 8 Monaten Erwachsenenniveau. Dagegen ist die Darmpassage eher beschleunigt. Hinzu kommt die Unreife der Sekretion und Aktivität der Gallen- und Pankreassäfte, was die Aufnahme lipidhaltiger Arzneimittel vermindern kann [32]. Zudem besteht eine komplexe Interaktion zwischen Nahrung und Arzneimittelresorption: Während z. B. Amoxicillin/Clavulansäure bei Nahrungsaufnahme verbessert resorbiert wird, sinkt die Resorption von Ampicillin bei gleichzeitiger Nahrungsaufnahme, die von z. B. Azithromycin bleibt unverändert [30]. Auch bei parenteraler Antiinfektivagabe kann die Pharmakokinetik bei Kindern verändert sein: So haben Neonaten und Kleinkinder eine verminderte Bioverfügbarkeit intramuskulär applizierter Substanzen, die Absorption durch die Haut kann dagegen erhöht sein [30, 32].

### Verteilung (Distribution).

Auch die Verteilung zeigt deutliche, stark vom Lebensalter abhängige Unterschiede zum Erwachsenen. So weisen Neonaten ein geringeres Gesamtprotein, Plasmaalbumin und -globulin sowie saures α1-Glycoprotein auf. Gleichzeitig ist der Körperwasseranteil im Vergleich deutlich erhöht. Dies kann zu veränderten Verteilungsvolumina z. B. für hydrophile Substanzen wie Aminoglykoside oder Glykopeptide bzw. Substanzen mit starker Plasmaproteinbindung wie Ceftriaxon führen. Mit zunehmendem Alter reduzieren sich diese Unterschiede zum Erwachsenen. Gerade bei sehr jungen und unreifen Kindern kann die Blut-Hirn-Schranke noch unvollständig ausgebildet sein. Infolge können Substanzen den Liquor erreichen, die ansonsten nicht liquorgängig sind [30, 32].

### Verstoffwechselung (Metabolismus).

Die im Vergleich zu Erwachsenen veränderte Fähigkeit zur Verstoffwechselung von Antiinfektiva kann je nach beteiligtem Enzymsystem zu niedrigeren oder höheren Plasmakonzentrationen bei Kindern, ähnlich wie bei Älteren, führen. Gerade bei Neu- und Frühgeborenen sind dabei viele der für Phase-I-Reaktionen wichtigen CYP-Enzyme noch nicht voll aktiv bzw. werden nur vermindert exprimiert. Gleiches gilt für wichtige Phase-II-Enzyme [30, 32].

### Ausscheidung (Elimination).

Die glomeruläre Filtrationsrate von Neonaten ist im Vergleich zu Erwachsenen deutlich reduziert und erreicht erst nach ca. 3 Monaten das Niveau von Erwachsenen – bei Frühgeborenen entsprechend später. Bei Säuglingen jenseits von 3 Monaten kann sie die GFR von Erwachsenen sogar deutlich übertreffen. Das kann klinische Auswirkungen v. a. auf Antiinfektiva haben, die in erster Linie renal ausgeschieden werden, wie z. B. Aminoglykoside, für die gezeigt werden konnte, dass mit zunehmendem Gestationsalter bei Neugeborenen < 7 Tagen die Plasmahalbwertszeit abnahm. Andererseits sollte bei der Gabe von Aminoglykosiden an Frühgeborene mit einem postkonzeptionellen Alter von < 34 Wochen beachtet werden, dass die Halbwertszeit verlängert ist [32].

## Diskussion

Die Behandelbarkeit von Infektionen mit Antiinfektiva ist eine der größten Errungenschaften der Medizin des 20. Jahrhunderts. Dabei sind der Einsatz von Antiinfektiva und das Problem der Resistenz gegen Antiinfektiva untrennbar miteinander verbunden, denn jeder Antiinfektivaeinsatz erzeugt evolutionären Druck auf die Pathogene. Die der Resistenzentwicklung zugrunde liegenden molekularen Prozesse sind an anderer Stelle ausführlich beschrieben [33].

Um der Entwicklung von Resistenzen keinen Vorschub zu leisten, ist es wichtig, Antiinfektiva so einzusetzen, dass die Erreger abgetötet werden. Je nach Substanz ist es dazu nötig, ausreichend hohe und/oder lang anhaltende Wirkspiegel im Vergleich zur MHK am Zielort zu erreichen. Hierzu ist neben der Kenntnis von MHK und Pharmakodynamik vor allem auch fundiertes Wissen zur Pharmakokinetik notwendig. Nur so ist es möglich, das Antiinfektivum wirksam zu dosieren. Auch heute noch werden Studien zur Pharmakokinetik vorwiegend an Erwachsenen mittleren Alters durchgeführt [2–5]. Das kann bei Patientengruppen mit im Vergleich zu dieser Gruppe veränderter Pharmakokinetik, wie Älteren und Kindern, dazu führen, dass nicht die beabsichtigten Wirkspiegel erreicht werden [19, 25–30].

Gerade bei älteren Patienten besteht zudem das Problem, dass die Antiinfektivagabe zu einer häufig vorbestehenden Multimorbidität und Polypharmazie hinzukommt [25]. Damit steigt das Risiko für eine Fehldosierung mit der Folge einer nicht ausreichenden therapeutischen Wirkung und Begünstigung der Selektion resistenter Klone einerseits und für eine Überdosierung mit erhöhter Wahrscheinlichkeit für Nebenwirkungen andererseits (Tab. 1 für Beispiele). Tatsächlich sind in den USA unerwünschte Wirkungen von Antiinfektiva für ca. 3,8 % der Hospitalisierungen ursächlich [15]. Eine Unterdosierung erhöht nicht nur direkt die Wahrscheinlichkeit der Entwicklung von Resistenzen, sondern auch eines klinischen Therapieversagens und/oder der Notwendigkeit einer Verlängerung, Umstellung oder Intensivierung der Therapie. Infolge steigen die Gesamtmenge des Medikamentenbedarfs und möglicherweise auch die Anzahl der verabreichten Antiinfektiva.

**Tab. 1:** Medikamenteninteraktionen, die die Absorption, Verstoffwechselung und Exkretion von Antiinfektiva beeinflussen. Beispiele für Medikamente, die häufig bei älteren Menschen verschrieben werden [17, 25, 35]

Substanzgruppen, die mit Antiinfektiva interagieren	Interaktion	Betroffene Antiinfektiva (Beispiele)
Protonenpumpeninhibitoren (PPIs)	Reduzierte Magensäure, erhöhter pH-Wert im Magen	Azithromycin, Erythromycin, Cefaclor, Ceftibuten, Itraconazol, Ketoconazol, Sulfonamide, Dapson, Pyrimethamin, Atazanavir
Diverse Medikamente (z. B. Fentanyl, Diazepam, Glipizid, Losartan, Omeprazol Nifedipin, Metoprolol, Timolol, Amidodaron, Citalopram, Carbamazepin, Topiramat)	Substanzabhängige Kompetition, Inhibition oder Induktion um/von CYP-P450-Enzymen	Makrolide, Fluorchinolone, Azole, antiretrovirale Medikamente, Rifampicin, Isoniacid

**Abb. 2:**
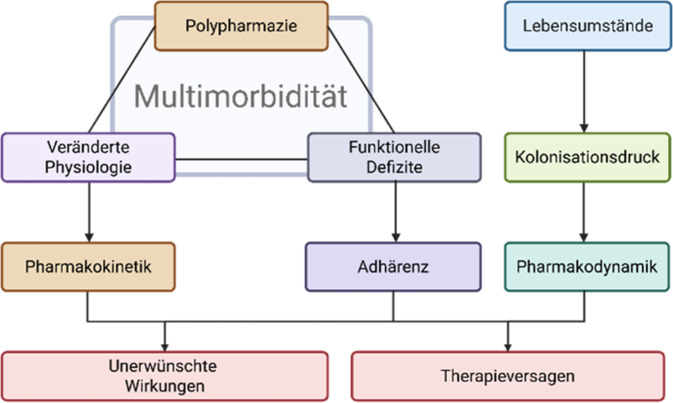
Faktoren, die bei der Dosierung von Antiinfektiva für ältere Menschen zu beachten sind, um unerwünschte Wirkungen und Therapieversagen zu vermeiden. Stark vereinfachte Zusammenhänge zwischen Pharmakokinetik, Pharmakodynamik, Adhärenz und den sie bestimmenden Faktoren

Neben den Herausforderungen durch veränderte PK/PD-Parameter und Polypharmazie ist auch die Adhärenz zu bedenken [17]. Bei Pflegeheimbewohnern verstärken sich die Effekte durch die hohe Dichte infektionsgefährdeter und infizierter Personen und die hohe Einsatzdichte von Antiinfektiva, was zu einem hohen Selektions- und Kolonisationsdruck führt [22, 25, 34]. Abb. 2 stellt dazu stark vereinfacht die Herausforderungen bei einer Antiinfektivatherapie insbesondere älterer Menschen dar.

## Fazit

Für eine erfolgreiche antiinfektive Therapie bei Personen, die sich von der üblichen Referenzgruppe der „Erwachsenen im mittleren Alter“ relevant unterscheiden, müssen die Besonderheiten in der Pharmakokinetik und Pharmakodynamik sowie mögliche Interaktionen mit bestehender Medikation berücksichtigt werden. Unterlässt man dies, droht eine Fehldosierung, die die Erregerelimination beeinträchtigt, die Entwicklung von Resistenzen begünstigt und zu klinischem Therapieversagen und Nebenwirkungen führt. Obwohl umfassende Übersichten auch zur Pharmakokinetik einzelner Substanzen existieren, bleibt die Anpassung der Dosis in der Praxis eine Herausforderung [15, 19, 25]. Das gilt vielleicht insbesondere für ältere Menschen, die, wenn keine starke Pflegebedürftigkeit vorliegt, leicht wie „normale Erwachsene“ behandelt werden. Daher sind eine sorgfältige Anamnese, die Abschätzung möglicher Interaktionen und eine bewusste Dosisfindung unerlässlich. Zudem bedarf es der intensiven Aufklärung der Patienten über die korrekte Applikation, mögliche Neben- und Wechselwirkungen und die Notwendigkeit der Einnahme der Medikamente gemäß Verordnung, um eine gute Adhärenz zu erreichen.

Die Vielfalt der stark substanz- und patientenabhängigen Effekte erfordert eine individuelle Behandlung, insbesondere bei pädiatrischen und älteren Patienten, bei denen spezifische Aspekte in der Therapie beachtet werden müssen: Welche Risiken für eine veränderte PK/PD im Vergleich zur „Normalgruppe“ bestehen? Welche Interaktionen können auftreten? Welche Effekte sind zu erwarten? Welche Effekte könnten überwiegen – werden die Spiegel eher erhöht oder erniedrigt sein? Wie kann man gegensteuern und das Monitoring sicherstellen? Wie erkennt man Nebenwirkungen oder Therapieversagen und wie reagiert man darauf? Daher ist die Berücksichtigung von PK/PD-Parametern gerade in diesen Gruppen ein essenzieller Bestandteil eines gelebten Antimicrobial/Antibiotic Stewardship (AMS/ABS).

## Data Availability

Alle im Rahmen dieser Studie gewonnenen oder analysierten Daten sind in dem vorliegenden Artikel enthalten.
